# Unruptured Aneurysm of Sinus of Valsalva Coexisting with the Large Ventricular Septal Defect and Severe Aortic Regurgitation in a Young Man

**DOI:** 10.1155/2015/396098

**Published:** 2015-07-07

**Authors:** Pouya Nezafati, Mohammad Hassan Nezafati, Hamid Hoseinikhah

**Affiliations:** ^1^Cardiac Surgery Research Committee, Mashhad University of Medical Sciences, Mashhad 9137913316, Iran; ^2^Department of Cardiac Surgery, Imam Reza Hospital, Mashhad University of Medical Sciences, Mashhad 9137913316, Iran

## Abstract

*Introduction*. Unruptured sinus of valsalva aneurysm (SVA) is a rare congenital anomaly, particularly, when it coexists with a ventricular septal defect (VSD) and aortic regurgitation due to the prolapse of the elongated aortic cusp into the VSD. In this report, we present the case of a 19-year-old young man with VSD challenging in spite of dyspnea and lower limb edema. *Presentation of Case*. Its diagnosis was made on the basis of transthoracic echocardiography results. Surgical management consisted of replacing the SVA with mechanical valve prosthesis. A Gore-Tex patch repaired the VSD. *Discussion*. In the follow-up periods, clinical and echocardiographic tests showed that the patient was in excellent status. *Conclusion*. SVA requires a surgical procedure due to its high risk of mortality in unoperated patients and a good safety of surgery.

## 1. Introduction

Sinus of valsalva aneurysms (SVA) is rare cardiac anomaly that may be congenital or, in rare cases, acquired and most commonly involves the right coronary sinus (90%) [1–4]. Congenital aneurysms are often caused by weakness at the junction of the aortic media and the annulus fibrosus [3]. The acquired forms are caused by infection, degenerative disease, or thoracic trauma. However, the majority of unruptured aneurysms do not produce symptoms of cardiac dysfunction until aneurysm rupture occurs [3, 4]. We report the case of a 19-year-old patient with unruptured SVA, which coexisted with a ventricular septal defect (VSD) challenging in spite of dyspnea and lower limb edema.

## 2. Case Report

**Figure 1:**
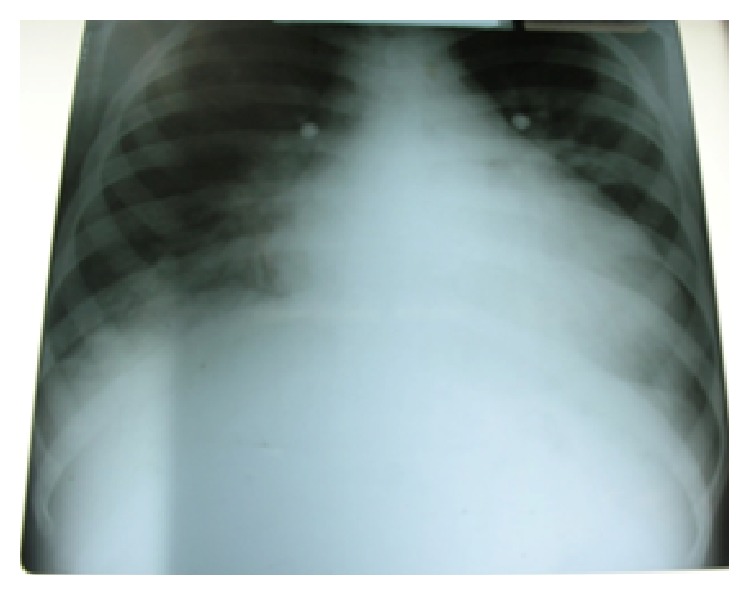
Chest X-ray showing the massive cardiomegaly.

A 19-year-old young man had a two-year history of exertional dyspnea, lower limb edema, periodic chest pain, and jaundice. The patient was in NYHA classes II and III with his symptoms progressively worsening over time. His past medical history indicated the successful balloon angioplasty and stenting for coarctation of the aorta (COA) at the age of 15, which left an arterial hypertension and no significant residual COA, and in the follow-up periods. His clinical evaluation based on the chest X-ray findings showed cardiomegaly, particularly, in the right ventricle and pulmonary congestion (Figure 1). Laboratory data showed that he had a mild increase in the serum creatinine (Cr), bilirubin (BIL), and nonalcoholic fatty liver disease (NAFLD). Furthermore, transthoracic echocardiographic examination (TTE) indicated that the patient had a large VSD and prolapse of the noncoronary cusp of the aorta into the VSD due to severe aortic regurgitation. Left ventricular (LV) ejection fraction (EF) index was about 40%, left ventricle end diastolic diameter (LVEDD) was 75 mm, and left ventricle end systolic diameter (LVESD) was 55 mm. The catheterization prior to surgery indicated that Qp/Qs was 2.2. Also his pulmonary artery pressure was estimated to be 60 mm Hg and the pulmonary artery pressure to aortic pressure ratio was 0.7.

**Figure 2:**
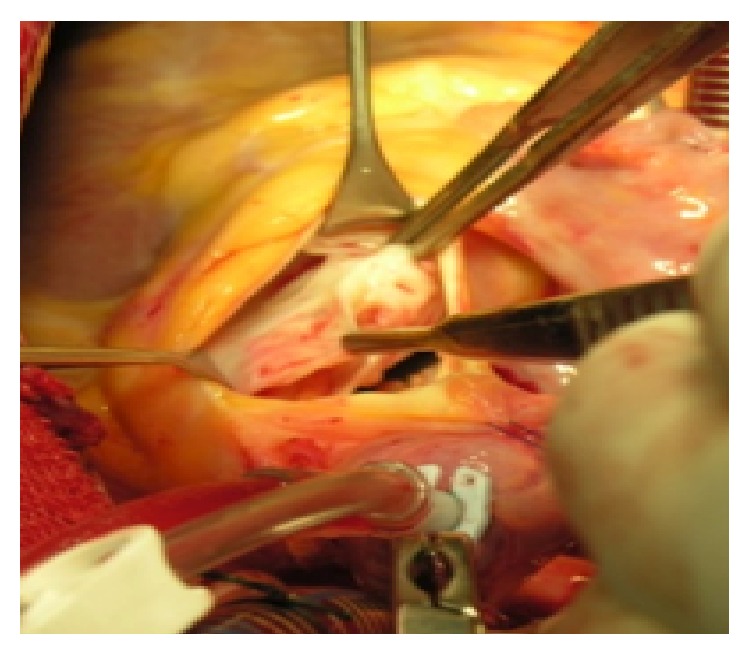
The aneurysm sac of the sinus of valsalva.

After his complete evaluation, surgery was recommended. After initiating the cardiopulmonary bypass machine and arresting the heart function, a significant aortic valve insufficiency with perforation of the right and left aortic cusps in the base segment was observed. In addition, prolapse of the noncoronary aortic cusp into the large subarterial VSD was seen. The right coronary sinus of valsalva showed an aneurysmal formation that was attached to the right ventricle with a short tract. Also, there was a mild dilation of the aortic root and the ascending aorta. The aortic leaflet was also thin and perforated, and its excision was sent for a further pathological examination (Figure 2). After removing the aneurysmal sac of the right coronary artery, the defect that was created in the right ventricle (RV) was closed with a small piece of patch. Furthermore, the VSD was repaired using a Gore-Tex patch and the aortic valve replacement was performed with a St. Jude mechanical aortic prosthetic valve. Weaning the patient from cardiopulmonary bypass was successful, and the patient was discharged 1 week later after the recovery. In the follow-up period, echocardiography tests showed that the patient had no residual VSD, and a good hemodynamic status of the prosthetic valve was also observed.

## 3. Discussion

An SVA is a rare congenital heart disease with incidences of 0.5% to 3% among all congenital heart diseases. SVA could occur in the right and left sinuses and in a noncoronary aortic sinus. The right coronary sinus is the most common type for this congenital anomaly with a prevalence of 75–90%. After the right coronary sinus, a noncoronary sinus type is more prevalent than the left coronary sinus. Rupture of SVA is five times higher in Asian population as compared with the Western population. Previous studies confirmed that SVA could occur at any age ranging from 2 to 74 years, the average being 39 years [5, 6].

Although the etiology of SVA is congenital, generally, a minority of this anomaly could be attributed due to trauma, infection, degeneration, and collagen vascular disease [7, 8]. The other congenital cardiac anomalies that coexist with SVA are VSD in about 30–60% of cases, which includes bicuspid aortic valve, aortic valve stenosis, pulmonary stenosis, COA, patent ductus arteriosus, atrial septal defect, and tricuspid insufficiency [9, 10]. Rupture of SVA might occur due to trauma or endocarditis or could be spontaneous. The most common site of perforation of SVA rupture is the RV in 60% of the cases, followed by right atrium (RA), left atrium (LA), and LV. Free perforation and extracardiac rupture into the pericardial cavity and pleural space are uncommon, but fetal diagnosis of SVA could be made with TTE and transesophageal echocardiogram with an accuracy up to 90% [5, 6, 11]. Although catheterism and angiography are the gold standards of modality for diagnosis of this disease, these are not performed routinely. Computed tomography (CT) and magnetic resonance imaging (MRI) also have excellent accuracies for diagnosis [12, 13].

The percutaneous closure (PC) has been a therapeutic intervention over the last 20 years; however this lesion has been historically repaired with surgery with the average survival time of about 3.9 years [14]. Reports reveal that PC in patients with severe aortic regurgitation could not be safe, effective, and practical [14]. It is often necessary that the aortic valve be repaired or replaced. Repair of SVA could be performed from the aorta or from the chamber where the aneurysm has been penetrated. In some complicated cases, this is done from both sides [15]. The operative mortality is less, close to1%, but, in individuals with SVA along with endocarditis, the mortality could reach 3.5% [16]. In the long-term follow-up for patients who underwent a surgery, survival rates after 5 and 10 years are about 97% and 82%, respectively. The postoperative prognosis appears to be better if the surgeon can avoid aortic valve replacement due to the subsequent prosthetic dysfunction [16].

In conclusion, SVA indicates an obvious surgical procedure owing to the high risk of death in unoperated patients and the low mortality rate due to surgery.
